# Shorter Dual Antiplatelet Therapy for Older Adults After Percutaneous Coronary Intervention: A Systematic Review and Network Meta-Analysis

**DOI:** 10.1001/jamanetworkopen.2024.4000

**Published:** 2024-03-28

**Authors:** Dae Yong Park, Jiun-Ruey Hu, Yasser Jamil, Michelle D. Kelsey, W. Schuyler Jones, Jennifer Frampton, Ajar Kochar, Wilbert S. Aronow, Abdulla A. Damluji, Michael G. Nanna

**Affiliations:** 1 Department of Medicine, Cook County Health, Chicago, Illinois; 2 Section of Cardiovascular Medicine, Yale School of Medicine, New Haven, Connecticut; 3 Department of Medicine, Yale-Waterbury Hospital, Waterbury, Connecticut; 4 Division of Cardiology, Duke University Medical Center, Durham, North Carolina; 5 Duke Clinical Research Institute, Durham, North Carolina; 6 Department of Cardiovascular Medicine, Brigham and Women’s Hospital, Harvard Medical School, Boston, Massachusetts; 7 Richard and Susan Smith Center for Outcomes Research in Cardiology, Beth Israel Deaconess Medical Center, Harvard Medical School, Boston, Massachusetts; 8 Department of Cardiology, Westchester Medical Center, Valhalla, New York; 9 Division of Cardiology, Johns Hopkins University School of Medicine, Baltimore, Maryland; 10 Inova Center of Outcomes Research, Inova Heart and Vascular Institute, Falls Church, Virginia

## Abstract

**Importance:**

The optimal duration of dual antiplatelet therapy (DAPT) for older adults after percutaneous coronary intervention (PCI) is uncertain because they are simultaneously at higher risk for both ischemic and bleeding events.

**Objective:**

To investigate the association of abbreviated DAPT with adverse clinical events among older adults after PCI.

**Data Sources:**

The Cochrane Library, Google Scholar, Embase, MEDLINE, PubMed, Scopus, and Web of Science were searched from inception to August 9, 2023.

**Study Selection:**

Randomized clinical trials comparing any 2 of 1, 3, 6, and 12 months of DAPT were included if they reported results for adults aged 65 years or older or 75 years or older.

**Data Extraction and Synthesis:**

The Preferred Reporting Items for Systematic Reviews and Meta-Analyses (PRISMA) reporting guideline was used to abstract data and assess data quality. Risk ratios for each duration of DAPT were calculated with alternation of the reference group.

**Main Outcomes and Measures:**

The primary outcome of interest was net adverse clinical events (NACE). Secondary outcomes were major adverse cardiovascular events (MACE) and bleeding.

**Results:**

In 14 randomized clinical trials comprising 19 102 older adults, no differences were observed in the risks of NACE or MACE for 1, 3, 6, and 12 months of DAPT. However, 3 months of DAPT was associated with a lower risk of bleeding compared with 6 months of DAPT (relative risk [RR], 0.50 [95% CI, 0.29-0.84]) and 12 months of DAPT (RR, 0.57 [95% CI, 0.45-0.71]) among older adults. One month of DAPT was also associated with a lower risk of bleeding compared with 6 months of DAPT (RR, 0.68 [95% CI, 0.54-0.86]).

**Conclusions and Relevance:**

In this systematic review and meta-analysis of different durations of DAPT for older adults after PCI, an abbreviated DAPT duration was associated with a lower risk of bleeding without any concomitant increase in the risk of MACE or NACE despite the concern for higher-risk coronary anatomy and comorbidities among older adults. This study, which represents the first network meta-analysis of this shortened treatment for older adults, suggests that clinicians may consider abbreviating DAPT for older adults.

## Key Points

**Question:**

What is the optimal duration of dual antiplatelet therapy (DAPT) in terms of net adverse clinical events, major cardiovascular events, and bleeding for older adults after percutaneous coronary intervention (PCI)?

**Findings:**

In this systematic review and network meta-analysis of 14 randomized clinical trials, no differences in net adverse clinical events and major adverse cardiovascular events were seen for 1, 3, 6, and 12 months of DAPT. However, 1 and 3 months of DAPT were each associated with lower risk of bleeding compared with 6 months of DAPT; in addition, 3 months of DAPT was associated with a lower risk of bleeding compared with 12 months of DAPT.

**Meaning:**

Despite the concern for higher ischemic risk among older adults, clinicians can consider abbreviating the duration of DAPT after PCI to reduce the risk of bleeding without increasing the risk of major adverse cardiovascular events.

## Teaser

This systematic review and network meta-analysis investigates the risk of adverse clinical events associated with different durations of dual antiplatelet therapy for older adults after percutaneous coronary intervention.

## Introduction

As the population ages, clinicians are more frequently caring for older adults undergoing percutaneous coronary intervention (PCI) and subsequently requiring dual antiplatelet therapy (DAPT).^1^ After a PCI, current guidelines recommend 6 months of DAPT, regardless of age, for stable ischemic heart disease and 12 months of DAPT for acute coronary syndrome (ACS).^2^ They also allow for an individualized strategy with discontinuation of aspirin after 1 to 3 months of DAPT in select cases.

However, a uniform approach to DAPT duration after a PCI across the age spectrum may be suboptimal. Older adults have been variably defined as 65 years of age or older to 75 years of age or older depending on the literature source, have more complex coronary anatomy, and are at the highest risk for future cardiovascular events, while simultaneously having the highest risk for bleeding complications.^3,4,5^ Despite the distinct anatomical and clinical risk factors and their potential implications for adverse outcomes associated with purinergic receptor P2Y, G-protein–coupled, 12 protein (P2Y12) inhibitors among older adults, dedicated studies on the duration of DAPT for older adults undergoing PCI remain limited.^6,7,8^ Therefore, we aimed to examine the association of abbreviated DAPT with adverse clinical events, major adverse cardiovascular events, and bleeding among older adults by performing a systematic review and network meta-analysis of randomized clinical trials (RCTs).

## Methods

All supporting data are available within the article and the supplementary materials. Our systematic review was conducted according to a published protocol available in Open Science Framework^9^ and is in adherence with the Preferred Reporting Items for Systematic Reviews and Meta-Analyses (PRISMA) reporting guideline.^10^ Our study was exempt from institutional review board because we used data from previously published articles only.

### Search Strategy and Inclusion Criteria

Two authors (D.Y.P. and J.-R.H.) systematically searched the literature via the following databases: Cochrane Library, Google Scholar, Ovid Embase, Ovid MEDLINE, PubMed, Scopus, and Web of Science Core Collection from the inception of each database to August 9, 2023. The search was formulated using adjudicated vocabulary and keywords with synonyms for DAPT, duration of treatment, PCI, and RCT, similar to those used by a previous study.^11^ Full search strategies for all databases are available in eTable 1 in Supplement 1. Once relevant studies were collected, the reference lists of each study were searched for additional relevant literature. Duplicate studies were excluded when citations were imported from the initial search into EndNote, version 20 software (Clarivate). Titles, abstracts, full manuscripts, and supplementary appendices were successively screened for eligibility, and selected studies were reexamined for accuracy by the corresponding author (M.G.N.). Conflicts were settled after a team discussion under the supervision of the corresponding author (M.G.N.).

Studies were included if they met the following criteria: (1) RCT; (2) comparison between any 2 of 1, 3, 6, and 12 months of DAPT; (3) reporting of outcomes associated with older adults; (4) follow-up duration of at least 9 months after index PCI; and (5) written in English. Because there was no study that exclusively enrolled older adults, data were extracted from the subgroup analyses of RCTs. We included older adult cutoffs based on individual trial definitions; most trials included an age cutoff of 65 years or older or 75 years or older (Table).^12,13,14,15,16,17,18,19,20,21,22,23,24,25^ Unification of the definition was not possible because we did not have access to individual patient-level data. When 2 or more studies using the same RCT data were found, the original article that preserved the effect of randomization was prioritized. For each selected trial, we used the Cochrane Collaboration’s tool (RoB1)^26^ to evaluate the risk of bias, and for each pooled outcome, we used the GRADE (Grading of Recommendations Assessment, Development and Evaluation) system to assess its quality.^27^

**Table.** Main Characteristics of the Included Trials

Trial	Year^a^	Country	ACS, %^b^	Age, y^c^	SAPT	Stent	Experimental arm		Control arm	
							No. of patients^d^	Duration of DAPT, mo	No. of patients^d^	Duration of DAPT, mo
Han et al,^12^ 2023 (HOST-IDEA)	2016-2021	South Korea	55.2	>65	Any^e^	Biodegradable or polymer-free SES	501	3	520	12
Valgimigli et al,^13^ 2021 (MASTER DAPT)	2017-2019	Multinational	48.3	≥75	Clopidogrel, aspirin	SES	1582	1	1572	6
Kim et al,^14^ 2020 (TICO)	2015-2018	South Korea	100	≥65	Ticagrelor	SES	582	3	603	12
Hahn et al,^15^ 2019 (SMART-CHOICE)	2014-2017	South Korea	58.2	≥75	Clopidogrel	EES, SES	791	3	743	12
Mehran et al,^16^ 2019 (TWILIGHT)	2015-2017	Multinational	64.8	≥65	Ticagrelor	Second-generation DES^f^	1841	3	1836	12
Watanabe et al,^17^ 2019 (STOPDAPT-2)	2015-2017	Japan	38.2	≥65	Clopidogrel	Cobalt-chromium EES	448	1	499	12
De Luca et al,^18^ 2019 (REDUCE)	2014-2016	Multinational	100	≥75	Aspirin	CD34+ antibody–coated SES	100	3	97	12
Franzone et al,^19^ 2019 (GLOBAL LEADERS)	2013-2015	Multinational	50.6	>75	Ticagrelor	BES	658	1	652	12
Hahn et al,^20^ 2018 (SMART-DATE)	2012-2015	South Korea	100	≥65	Aspirin	ZES, EES, BES	596	6	603	12
Hong et al,^21^ 2016 (IVUS-XPL)	2010-2014	South Korea	49.0	>65	Aspirin	EES	307	6	343	12
Schulz-Schüpke et al,^22^ 2015 (ISAR-SAFE)	2008-2014	Multinational	40.7	≥67.2	Aspirin	EES, SES, ZES, BES, PES	999	6	1002	12
Han et al,^23^ 2016 (I-LOVE-IT 2)	2012-2013	China	81.8	≥65	Aspirin	Biodegradable-polymer SES	304	6	283	12
Kim et al,^24^ 2012 (RESET)	2009-2010	South Korea	54.6	≥65	Aspirin	ZES	477	3	487	12
Gwon et al,^25^ 2012 (EXCELLENT)	2008-2009	South Korea	48.5	≥65	Aspirin	EES, SES	341	6	335	12

Abbreviations: ACS, acute coronary syndrome; BES, biolimus-eluting stent; DAPT, dual antiplatelet therapy; DES, drug-eluting stent; EES, everolimus-eluting stent; PES, paclitaxel-eluting stent; SAPT, single antiplatelet therapy; SES, sirolimus-eluting stent; ZES, zotarolimus-eluting stent.

^a^ Year of enrollment.

^b^ Mean of the percentage of ACS in abbreviated and standard DAPT groups.

^c^ Cutoff defining older adults in each of the trials.

^d^ Sample size of the older adult population.

^e^ Any antiplatelet agent in the trial at the discretion of the ordering physician: aspirin (64.1%), clopidogrel (33.7%), ticagrelor (1.9%), and prasugrel (0.3%).

^f^ Second-generation DES: durable polymer cobalt-chromium EES, durable polymer platinum-chromium EES, durable polymer ZES, durable polymer cobalt-chromium SES, biodegradable polymer DES, polymer-free DES, bioresorbable vascular scaffold, sirolimus-eluting self-apposing stent, and tacrolimus-eluting carbostent.

### Data Acquisition and Outcomes of Interest

The following information was collected from each study: acronym of the trial, year of enrollment, country in which the study was conducted, percentage of patients with ACS, single antiplatelet therapy (SAPT), types of stents deployed, and sample sizes. Baseline characteristics of patients were also gathered for comparison. The primary outcome of interest was net adverse clinical events (NACE). Secondary outcomes included major adverse cardiovascular events (MACE) and bleeding.

### Statistical Analysis

To maintain consistency across all studies, we manually calculated risk ratios (RRs) from all selected RCTs. Zero-cell correction was unnecessary because prespecified outcomes occurred at least once in all the studies. After collection of the outcomes, a frequentist network meta-analysis with random-effects model was conducted to calculate pooled estimates by alternating the reference group. Node-splitting analysis was performed to evaluate for inconsistencies between direct and indirect estimates. The τ^2^ and *I*^2^ values were used to assess for heterogeneity in the network models. P-scores for each duration of DAPT were calculated for each outcome and were interpreted only when the network meta-analysis showed significant differences among the different durations of DAPT. P-scores signify the mean extent of certainty that the respective duration of DAPT was better than other durations of DAPT averaged over all denominators with equal weights.^28^ In essence, a P-score of 0 represents the worst and a P-score of 1 represents the best. Net clinical benefit was illustrated by graphing bleeding against MACE for different durations of DAPT. A sensitivity analysis including only trials that defined older adults as 65 years or older (or similar), in contrast to 75 years or older, was performed. A sensitivity analysis including trials that defined older adults as 75 years or older could not be performed because of the scarcity of such trials. Another sensitivity analysis of trials that reported major bleeding, defined by Bleeding Academic Research Consortium (BARC) type 3 or greater bleeding or Thrombolysis In Myocardial Infarction (TIMI) major bleeding, was conducted. Frequentist network meta-analysis was performed using the meta and netmeta packages in R, version 4.2.3 (R Project for Statistical Computing).

## Results

### Study and Patient Characteristics

**Figure 1.**
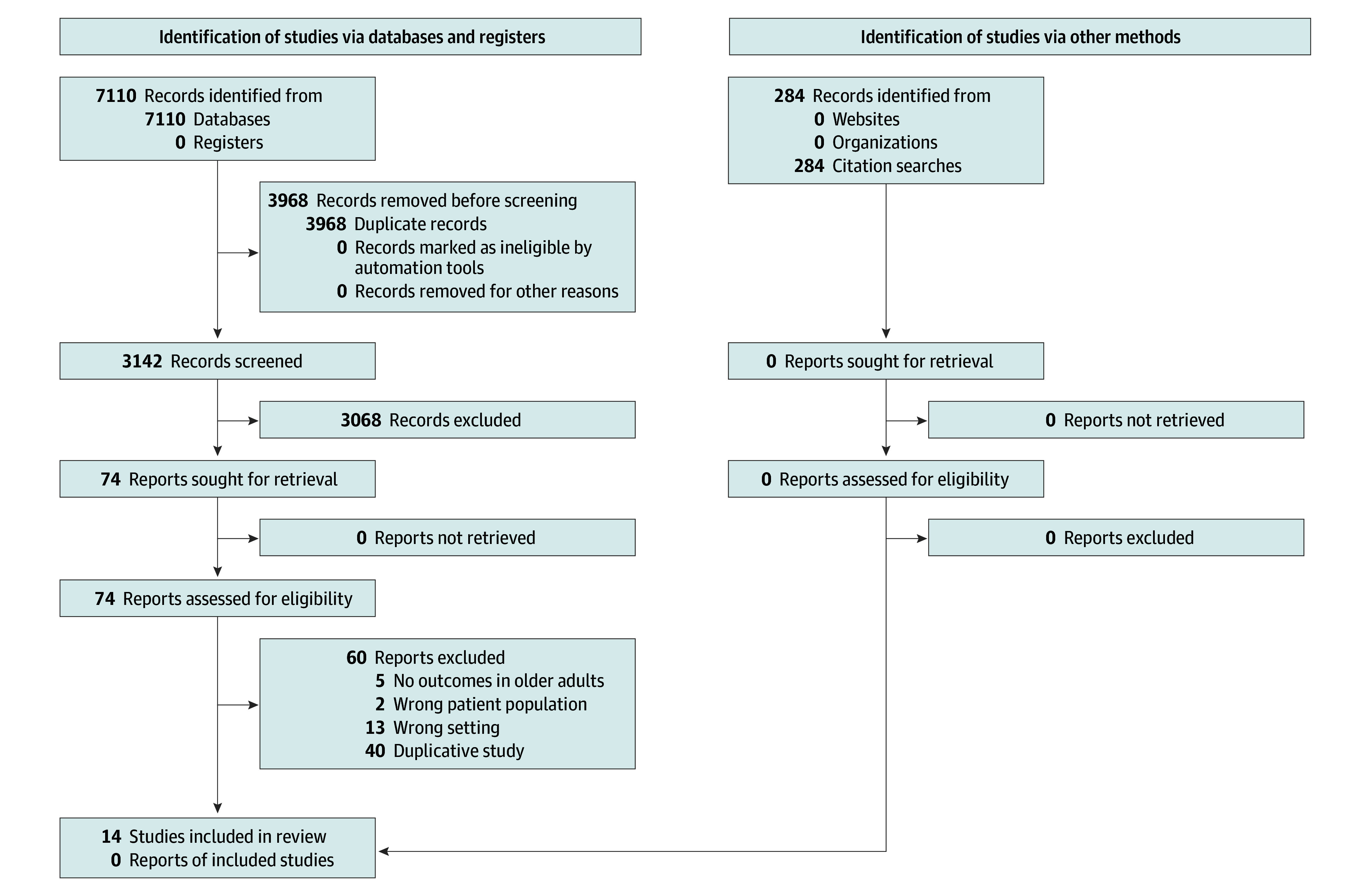
Preferred Reporting Items for Systematic Reviews and Meta-Analyses (PRISMA) Flow Diagram of the Meta-Analysis

**Figure 2.**
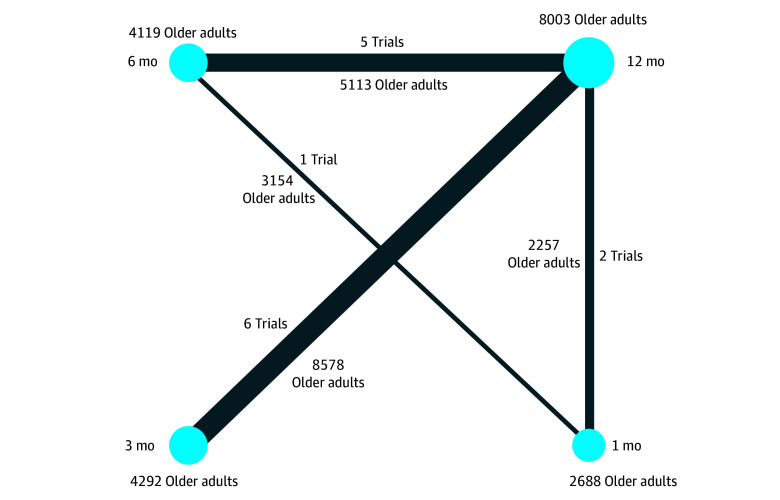
Network Plot of the Included Randomized Clinical Trials The network plot illustrates the number of trials and older adults among trials that compared 1 month, 3 months, 6 months, and 12 months of dual antiplatelet therapy. The sizes of the blue circles and blue lines are proportional to the total sample size and number of relevant trials, respectively.

A total of 14 RCTs^12,13,14,15,16,17,18,19,20,21,22,23,24,25^ with a cumulative sample size of 19 102 older adults were included in our systematic review (Figure 1). Six trials,^12,14,15,16,18,24^ which included 8578 older adults (44.9%), compared 3 months of DAPT with 12 months of DAPT (Figure 2). Five trials,^20,21,22,23,25^ which included 5113 older adults (26.8%), compared 6 months of DAPT with 12 months of DAPT. Two trials,^17,19^ which included 2257 older adults (11.8%), compared 1 month of DAPT with 12 months of DAPT. One trial,^13^ which included 3154 older adults (16.5%), compared 1 month of DAPT with 6 months of DAPT. The years of recruitment ranged from 2008 to 2021 (Table).^12,13,14,15,16,17,18,19,20,21,22,23,24,25^ Nine^12,14,15,17,20,21,23,24,25^ of the 14 trials were exclusively conducted in Asia, with 7^12,14,15,20,21,24,25^ of them conducted in South Korea. The remaining 5 trials^13,16,18,19,22^ were multinational studies that included diverse countries from the Americas, Europe, and Asia. Three trials^14,18,20^ exclusively enrolled patients with ACS, whereas the remaining 11 trials^12,13,15,16,17,19,21,22,23,24,25^ enrolled variable percentages of patients with ACS. The percentages of patients with ACS in the overall trial populations for the selected trials ranged from 38.2% to 100%. Assuming identical percentages of patients with ACS in the older adult population, 11 722 of the 19 102 older adults (61.4%) underwent a PCI for ACS. One trial^19^ defined older adults as older than 75 years, 2 trials^12,21^ defined older adults as older than 65 years, and 7 trials^14,16,17,20,23,24,25^ used 65 years of age or older as the cutoff. Two trials^12,19^ defined older adults as older than 75 years and as older than 65 years, respectively. Finally, 1 trial^22^ reported subgroup analysis among older adults 67.2 years of age or older. Seven trials^18,20,21,22,23,24,25^ used aspirin as SAPT, followed by 3 trials^14,16,19^ that used ticagrelor as SAPT and 2 trials^15,17^ that used clopidogrel as SAPT. One trial^13^ used both aspirin and clopidogrel, and another trial^12^ left the choice up to the ordering physicians, who predominantly used aspirin (64.1%), followed by clopidogrel (33.7%). The type of stents and baseline characteristics of patients were widely variable among the trials (Table^12,13,14,15,16,17,18,19,20,21,22,23,24,25^; eTable 2 in Supplement 1). The descriptions of these characteristics are from both the younger and older adults combined in each of the trials, as characteristics specific to only the older adult population were not available. The definitions of NACE, MACE, and bleeding also differed from one trial to another (eTable 3 in Supplement 1).

### Assessment of Biases

The risk of bias was mostly low in the selected trials, except for performance bias, which was high in 9 trials^12,14,15,17,18,20,21,24,25^ due to the open-label design (eTable 4 in Supplement 1). The quality of pooled outcomes was moderate to high (eTable 5 in Supplement 1). Heterogeneity observed in the frequentist network models ranged from none to moderate (eTable 6 in Supplement 1). The results of the node-splitting analysis of inconsistency can be found in eTable 7 and the eFigure in Supplement 1. A test of inconsistency demonstrated no statistically significant inconsistency within designs and between designs for all outcomes (eTable 8 in Supplement 1).

### Clinical Outcomes

**Figure 3.**
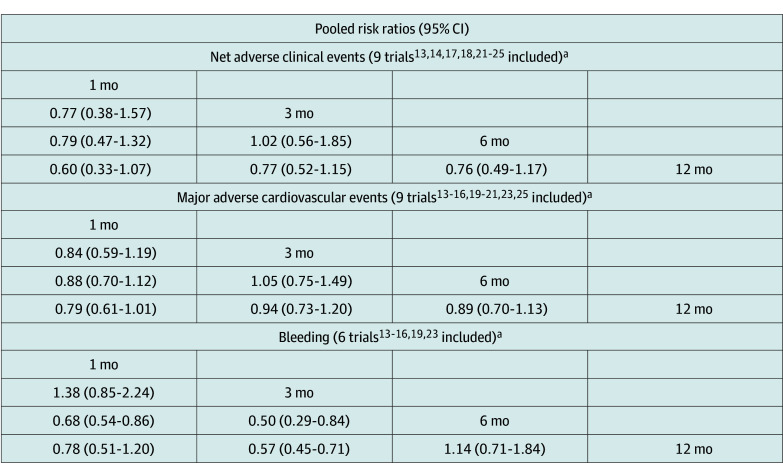
Pooled Risk Ratios of Frequentist Network Meta-Analysis for Each Outcome ^a^The duration of dual antiplatelet therapy at the rightmost column serves as the reference group for the respective column.

**Figure 4.**
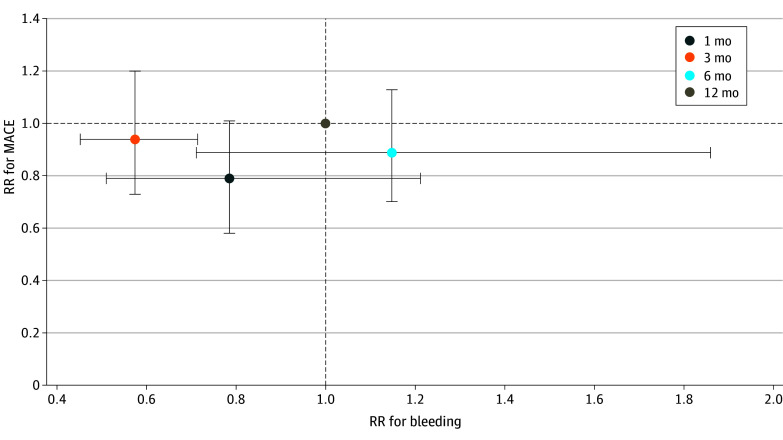
Net Clinical Benefit of Each Duration of Dual Antiplatelet Therapy for Older Adults Risk ratios (RRs) for different durations of dual antiplatelet therapy are plotted in comparison with 12 months of dual antiplatelet therapy (reference). Vertical error bars indicate the 95% CIs of the RRs for major adverse cardiovascular events (MACEs), and horizontal error bars indicate the 95% CIs of the RRs for bleeding.

No difference in the risk of NACE was observed among 1, 3, 6, and 12 months of DAPT (Figure 3).^13,14,15,16,17,18,19,20,21,22,23,24,25^ Similarly, no difference in the risk of MACE was observed among the 4 different durations of DAPT (1 vs 12 months: RR, 0.79 [95% CI, 0.61-1.01]; 3 vs 12 months: RR, 0.94 [95% CI, 0.73-1.20]; 6 vs 12 months: RR, 0.89 [95% CI, 0.70-1.13]). However, 1 month of DAPT was associated with a lower risk of bleeding (RR, 0.68 [95% CI, 0.54-0.86]; *P* = .001) compared with 6 months of DAPT. Three months of DAPT was associated with a lower risk of bleeding compared with 6 months of DAPT (RR, 0.50 [95% CI, 0.29-0.84]; *P* < .001) and 12 months of DAPT (RR, 0.57 [95% CI, 0.45-0.71]; *P* = .009) among older adults. No difference in the risk of bleeding was seen between 1 and 3 months of DAPT or between 6 and 12 months of DAPT. For the outcome of NACE, the P-score was highest for 1 month of DAPT, followed by 3 months, 6 months, and 12 months of DAPT (eTable 9 in Supplement 1). Similarly, the P-score was also highest for 1 month of DAPT for MACE, followed by 3 months, 6 months, and 12 months of DAPT. For bleeding, the P-score was highest for 3 months of DAPT, followed by 1 month, 6 months, and 12 months. The net clinical benefit favored 3 months of DAPT for having a lower risk of bleeding while having a nonsignificant risk of MACE in comparison with 12 months of DAPT (Figure 4). The cumulative event rates for all the DAPT durations and the absolute risk reductions at shorter durations of DAPT can be found in eTables 10 and 11 in Supplement 1.

### Sensitivity Analysis

Sensitivity analysis including trials that defined older adults as 65 years of age or older showed a lower risk of NACE (RR, 0.64 [95% CI, 0.46-0.91]; *P* = .01) and bleeding (RR, 0.57 [95% CI, 0.45-0.71]; *P* < .001) with 3 months vs 12 months of DAPT (eTable 12 in Supplement 1). Outcomes with 1 month of DAPT were unavailable because of the absence of trials that set the experimental group as 1 month of DAPT and defined older adults as 65 years of age or older. Sensitivity analysis of trials that reported major bleeding showed a lower risk of major bleeding with 3 months of DAPT compared with 12 months of DAPT (RR, 0.48 [95% CI, 0.24-0.94; *P* = .03) (eTable 13 in Supplement 1).

## Discussion

The optimal duration of DAPT for older adults remains controversial because older adults are simultaneously at higher risk for ischemic events and bleeding events and the association of DAPT duration with major events is not homogeneous across the lifespan. Current guidelines on coronary artery revascularization do not provide age-specific recommendations for the duration of DAPT after a PCI.^2^ To address this clinical uncertainty, the present study represents, to our knowledge, the first network meta-analysis of shorter-duration DAPT after a PCI among older adults. We found that an abbreviated DAPT duration of 1 or 3 months was associated with a lower risk of bleeding compared with 6 months, without any concomitant increase in the risk of MACE or NACE, despite a higher risk of complex coronary anatomy and increased calcification, comorbidities, and vascular tortuosity among older adults,^29^ features that traditionally make clinicians wary of abbreviating DAPT. A shortened DAPT duration of 3 months was also associated with a lower risk of bleeding compared with 12 months, without any concomitant increase in the risk of MACE or NACE.

The present meta-analysis extends to the 3-month and 1-month mark a pattern that has previously been demonstrated at the 12-month mark and the 6-month mark. In the DAPT score, which clinicians use to decide whether to prolong the duration of DAPT after 12 months, age is the only factor that carries negative points (discouraging prolonged DAPT duration), with being 75 years of age or older carrying −2 points and being 65 to 74 years of age carrying −1 point.^30^ In the prior major meta-analysis of DAPT duration among older adults, RCTs of short-term DAPT, defined as either 3 or 6 months, were compared with RCTs of long-term DAPT, defined as either 12 or 24 months.^31^ The primary outcome was the 12-month rate of a composite of myocardial infarction (MI), definite or probable stent thrombosis, or stroke, while the major secondary outcome was the 12-month rate of major bleeding. The authors found that shorter DAPT was noninferior to longer DAPT in terms of the ischemic composite of MI, stent thrombosis, or stroke for older adults (≥65 years) but was inferior for younger patients. Furthermore, shorter DAPT was associated with a reduced risk of bleeding compared with longer DAPT, but this benefit was statistically significant only for older adults. The key picture that emerges from these data is that for older adult patients, at longer durations of DAPT, the bleeding risk exceeds the thrombotic risk. Our meta-analysis, which focuses on comparing DAPT durations within 12 months, indicates that for older adults, reducing DAPT duration from 12 to 3 months is associated with a lower bleeding risk without increased MACE. The broader message underscored by the present meta-analysis and previous meta-analyses is that older adults do not benefit from a one-size-fits-all approach when it comes to duration of DAPT.

Ascertainment of the optimal duration of DAPT is especially important for older adults because they are simultaneously at increased risk for thrombotic events and bleeding events. All 3 components of Virchow’s triad are disrupted with aging, due to progressive loss of venous structural integrity, endothelial dysfunction, changes in the microcirculation,^32,33^ and upregulation of procoagulant coagulation factors (FV, FVII, FVIII, and FIX), thrombin, the von Willebrand factor, and d-dimer.^32,33^ This disruption is further compounded by the development of acquired prothrombotic states, such as cancer, autoimmune disorders, obesity, diabetes, or sedentary lifestyles, and states of abnormal blood flow, such as valvular stenosis and atrial fibrillation. At the same time, aging is associated with a decrease in platelet count, platelet turnover, and platelet function. In at least 1 study, among patients with ACS, age was associated with decreased adenosine diphosphate–induced platelet aggregation and decreased platelet activation, as indicated by decreased integrin αIIbβ3 activation and P-selectin exposure.^34^ For these reasons, older adults are at higher risk of bleeding events. This risk is further compounded by the presence of frailty and low body weight in the older adult population, potentially resulting in differences in the effect of standard antiplatelet and anticoagulant medications. When considering these competing pathophysiological processes in older adults, it is especially important to clarify the tradeoffs associated with each duration of DAPT, as our present network meta-analysis seeks to address. Within that context, our results suggest that the risk-benefit balance appears to weigh in favor of abbreviating the duration of DAPT whenever feasible to reduce the risk of bleeding.

Our study adds insight to the literature in several ways. At the time of the previous conventional meta-analysis of DAPT duration for older adults,^31^ the results of the TICO (Ticagrelor Monotherapy vs Ticagrelor With Aspirin in Patients With Acute Coronary Syndrome After Percutaneous Coronary Intervention) trial,^14^ the MASTER DAPT (Management of High-Bleeding-Risk Patients Post Bioresorbable Polymer Coated Stent Implantation With an Abbreviated Versus Prolonged DAPT Regimen) trial,^13^ the HOST-IDEA (Harmonizing Optimal Strategy for Treatment of Coronary Artery Stenosis–Coronary Intervention With Next-Generation Drug-Eluting Stent Platforms and Abbreviated Dual Antiplatelet Therapy) trial,^12^ the SMART-CHOICE (Comparison Between P2Y12 Antagonist Monotherapy and Dual Antiplatelet Therapy in Patients Undergoing Implantation of Coronary Drug-Eluting Stents) trial,^15^ the TWILIGHT (Ticagrelor With Aspirin or Alone in High-Risk Patients After Coronary Intervention) trial,^16^ the STOPDAPT-2 (Short and Optimal Duration of Dual Antiplatelet Therapy After Everolimus-Eluting Cobalt-Chromium Stent: A Randomized Multicenter Trial) trial,^17^ and the SMART-DATE (Smart Angioplasty Research Team: Safety of 6-Month Duration of Dual Antiplatelet Therapy After Percutaneous Coronary Intervention in Patients With Acute Coronary Syndromes) trial^20^ had not yet been published. The previous meta-analysis included 2 studies that were included in our network meta-analysis—the RESET (Real Safety and Efficacy of 3-Month Dual Antiplatelet Therapy Following Endeavor Zotarolimus-Eluting Stent Implantation) trial^24^ and the EXCELLENT (Efficacy of Xience/Promus Versus Cypher to Reduce Late Loss After Stenting) trial^25^—as well as 4 studies that were not included in our meta-analysis, the PRODIGY (Prolonging Dual Antiplatelet Treatment After Grading Stent-Induced Intimal Hyperplasia Study) trial,^35^ the OPTIMIZE (Optimized Duration of Clopidogrel Therapy Following Treatment With the Endeavor Zotarolimus-Eluting Stent in Real-World Clinical Practice) trial,^36^ the SECURITY (Second Generation Drug-Eluting Stent Implantation Followed by 6- Versus 12-Month Dual Antiplatelet Therapy) trial,^37^ and the ITALIC (Is There a Role for Triple Antiplatelet Therapy in Patients With Acute Coronary Syndromes With Indication for Percutaneous Coronary Intervention?) trial.^38^ The latter 4 studies did not meet the criteria for inclusion in our study because the control arms of the PRODIGY trial^35^ and the ITALIC trial^38^ were 24 months of DAPT, whereas the SECURITY trial^37^ and the OPTIMIZE trial^36^ did not report subgroup results for older adults. Our study further adds novelty because it is a network meta-analysis, which allows for the estimation of relative treatment effects of multiple DAPT durations that have not been directly compared head to head in individual trials, in contrast to a conventional meta-analysis approach, whereby DAPT duration of 1 or 3 months would be analyzed as a homogenous intervention and DAPT duration of 6 or 12 months would be analyzed as a homogenous intervention.

Our study was focused on ascertaining the optimal duration of DAPT; the optimal choice of subsequent SAPT remains a matter of debate. The included RCTs differed on the agent used for SAPT; thus, the choice of agent for post-DAPT SAPT remains a source of heterogeneity. A meta-analysis showed that compared with DAPT, both aspirin monotherapy and P2Y12 inhibitor monotherapy reduced major bleeding to a similar degree, but patients receiving post-DAPT aspirin monotherapy had a higher risk of MI compared with patients receiving post-DAPT P2Y12 inhibitor monotherapy.^39^ A recent scientific statement from the American Heart Association endorsed clopidogrel as the preferred P2Y12 inhibitor for older patients with ACS due to clopidogrel’s lower bleeding profile compared with ticagrelor or prasugrel, while leaving ticagrelor for those patients with the highest ischemic risk.^40^ Further studies will be required to confirm the optimal SAPT for older adults.

### Limitations

Our study should be interpreted in light of some limitations. First, all data were collected from published trials, which precludes the possibility of more granular subgroup analyses that would be possible with patient-level data. Information about genetic differences in metabolization of P2Y12 inhibitors are not available. Second, the included trials have variable compositions of clinical presentation, patient and procedural characteristics, stent types, and ancillary imaging, each of which may act as a confounder. There were different definitions of older adults across studies, although results remained consistent when those trials were considered separately. Third, the definitions of MACE, NACE, and bleeding were variable. For example, among the 6 of 14 trials that reported major bleeding, 3 reported BARC bleeding, 2 reported TIMI bleeding, and 1 reported major or nonmajor clinically relevant bleeding. Among the 9 of 14 trials that reported MACE, the definition of MACE was a composite of different combinations of all-cause mortality, MI, stroke, thrombosis, and target vessel revascularization. The MASTER DAPT,^13^ TICO,^14^ SMART-CHOICE,^15^ STOPDAPT-2,^17^ SMART-DATE,^20^ GLOBAL LEADERS,^19^ TWILIGHT,^16^ and EXCELLENT^25^ studies included stroke in their definition of MACE, thus including major adverse cerebral events, whereas the IVUS-XPL (Impact of Intravascular Ultrasound Guidance on the Outcomes of Xience Prime Stents in Long Lesions) trial^21^ and the I-LOVE-IT 2 (Is There a Life for DES After Discontinuation of Clopidogrel: Multicenter Study of the Endeavor Zotarolimus-Eluting Stent in Uncertain DES Candidates) trial^23^ did not. There was similar heterogeneity in the 9 of 14 trials that reported NACE. Fourth, many of the trials were open-label, making them susceptible to performance bias. Fifth, medication compliance and rates of frailty among the older adults were not available. Sixth, the choice of post-DAPT SAPT used in the group that received abbreviated DAPT durations differed across trials. Seventh, we had limited data on bleeding risk, such as baseline hemoglobin level and other hematologic pathologies, and geriatric-specific syndromes, such as frailty, that may influence the risk-benefit ratio for shorter vs longer DAPT durations.

## Conclusion

In this systematic review and meta-analysis of different durations of DAPT for older adults, we found that an abbreviated DAPT duration of 1 or 3 months was associated with a lower risk of bleeding without requiring a sacrifice in the rates of MACE or NACE. Our study should be confirmed by future patient-level meta-analyses or RCTs, which would also allow for investigation of subgroups of specific clinical phenotypes of older adults who would benefit most from an abbreviated DAPT strategy and those who may require a longer DAPT strategy.

## Notes

Supplement 1. **eTable 1.** Search Strategy Used in This Study **eTable 2.** Baseline Demographics of the Selected Trials **eTable 3.** Definition of Outcomes Used in the Trials **eTable 4.** Risk of Bias in the Selected Studies Using the Cochrane Risk Assessment Tool **eTable 5.** Grading of Recommendations, Assessment, Development and Evaluations (GRADE) Criteria for Each Outcome **eTable 6.** Heterogeneity Assessment in Network Meta-Analysis **eTable 7.** Node-Splitting Analysis of Inconsistency for Each Outcome **eTable 8.** Test of Inconsistency Between Designs **eTable 9.** P-Scores of Each Duration of Dual Antiplatelet Therapy **eTable 10.** Cumulative Event Rates at Different Durations of Dual Antiplatelet Therapy **eTable 11.** Absolute Risk Differences at Shorter Durations of Dual Antiplatelet Therapy **eTable 12.** Sensitivity Analysis Only Including Trials That Defined Older Adults as Age ≥65 Years **eTable 13.** Sensitivity Analysis Only Including Trials That Reported Major Bleeding in Older Adults **eFigure.** Node-Splitting Analysis of Inconsistency for Each Outcome
eReferences.

Supplement 2.
Data Sharing Statement
