# Supplementary material for: Shorter Dual Antiplatelet Therapy for Older Adults After Percutaneous Coronary Intervention: A Systematic Review and Network Meta-Analysis
Source: JAMA Netw Open. 2024 Mar 28;7(3):e244000. doi: 10.1001/jamanetworkopen.2024.4000 (PMC10979312; doi:10.1001/jamanetworkopen.2024.4000)
Supplement: Supplement 1. — eTable 1. Search Strategy Used in This Study eTable 2. Baseline Demographics of the Selected Trials eTable 3. Definition of Outcomes Used in the Trials eTable 4. Risk of Bias in the Selected Studies Using the Cochrane Risk Assessment Tool eTable 5. Grading of Recommendations, Assessment, Development and Evaluations (GRADE) Criteria for Each Outcome eTable 6. Heterogeneity Assessment in Network Meta-Analysis eTable 7. Node-Splitting Analysis of Inconsistency for Each Outcome eTable 8. Test of Inconsistency Between Designs eTable 9. P-Scores of Each Duration of Dual Antiplatelet Therapy eTable 10. Cumulative Event Rates at Different Durations of Dual Antiplatelet Therapy eTable 11. Absolute Risk Differences at Shorter Durations of Dual Antiplatelet Therapy eTable 12. Sensitivity Analysis Only Including Trials That Defined Older Adults as Age ≥65 Years eTable 13. Sensitivity Analysis Only Including Trials That Reported Major Bleeding in Older Adults eFigure. Node-Splitting Analysis of Inconsistency for Each Outcome eReferences. [file jamanetwopen-e244000-s001.pdf]

Supplementary Online Content

Park DY, Hu JR, Jamil Y, et al. Shorter dual antiplatelet therapy for older adults after percutaneous coronary intervention: a systematic review and network meta-analysis. *JAMA Netw Open.* 2024;7(3):e244000. doi:10.1001/jamanetworkopen.2024.4000

eTable 1. Search Strategy Used in This Study

eTable 2. Baseline Demographics of the Selected Trials

eTable 3. Definition of Outcomes Used in the Trials

eTable 4. Risk of Bias in the Selected Studies Using the Cochrane Risk Assessment Tool

eTable 5. Grading of Recommendations, Assessment, Development and Evaluations (GRADE) Criteria for Each Outcome

eTable 6. Heterogeneity Assessment in Network Meta-Analysis

eTable 7. Node-Splitting Analysis of Inconsistency for Each Outcome

eTable 8. Test of Inconsistency Between Designs

eTable 9. P-Scores of Each Duration of Dual Antiplatelet Therapy

eTable 10. Cumulative Event Rates at Different Durations of Dual Antiplatelet Therapy

eTable 11. Absolute Risk Differences at Shorter Durations of Dual Antiplatelet Therapy

eTable 12. Sensitivity Analysis Only Including Trials That Defined Older Adults as Age ≥65 Years

eTable 13. Sensitivity Analysis Only Including Trials That Reported Major Bleeding in Older Adults

eFigure. Node-Splitting Analysis of Inconsistency for Each Outcome

eReferences.

This supplementary material has been provided by the authors to give readers additional information about their work.

eTable 1. Search Strategy Used in This Study

Database	Search Strategy
**Cochrane Library**	#1 ((duration or length or timing or time or months or reduce* or alterat*) near/3 (dose* or therapy* or treatment* or medication* or DAPT or "dual anti-platelet")):ti,ab OR (de-escalat* or "prescribing practice*" or short-term or abbreviat*):ti,ab OR (duration or length or timing or time or month*):ti<br>#2 ((antiplatelet or anti-platelet) near/3 (dual or combination) near/3 (intervention* or therap*)):ti,ab or ((Aspirin or "Acetylsalicylic acid") and (clopidogrel or plavix or prasugrel or effient or ticagrelor or brilinta or "P2Y12 inhibitor*")):ti,ab or (DAPT):ti,ab<br>#3 ("percutaneous coronary" near/3 (interven* or revascular*)):ti,ab or (PCI):ti,ab or ((balloon or stent*) near/3 angioplast*):ti,ab or (("drug coated" or "drug eluting" or "drug releasing") near/3 stent*):ti,ab<br>#4 #1 and #2 and #3
**Ovid Embase**	1 exp percutaneous coronary intervention/<br>2 exp drug eluting stent/<br>3 (percutaneous coronary adj3 (interven* or revascular*)).tw,kf.<br>4 PCI.ti,ab.<br>5 ((balloon or stent*) adj3 angioplast*).tw,kf.<br>6 ((drug coated or drug eluting or drug releasing) adj3 stent*).tw,kf.<br>7 1 or 2 or 3 or 4 or 5 or 6<br>8 exp dual antiplatelet therapy/<br>9 ((antiplatelet or anti-platelet) adj3 (dual or combination) adj3 (intervention* or therap*)).tw,kf.<br>10 DAPT.ti,ab.<br>11 ((Aspirin or Acetylsalicylic acid) and (clopidogrel or plavix or prasugrel or effient or ticagrelor or brilinta or P2Y12 inhibitor*)).tw,kf.<br>12 8 or 9 or 10 or 11<br>13 7 and 12<br>14 exp randomized controlled trial/<br>15 exp single blind procedure/<br>16 double blind procedure/<br>17 crossover procedure/<br>18 (random* or factorial* or crossover* or placebo* or assign* or allocat* or volunteer*).tw,kf.<br>19 (cross adj1 over).tw,kf.<br>20 ((double or triple or single) adj1 (mask* or blind*)).tw,kf.<br>21 14 or 15 or 16 or 17 or 18 or 19 or 20<br>22 13 and 21<br>23 treatment duration/<br>24 ((duration or length or timing or time or months or reduce* or alterat*) adj3 (dose* or therap* or treatment* or medication* or DAPT or dual anti-platelet)).tw,kf.<br>25 (duration or length or timing or time or month*).ti.<br>26 (de-escalat* or prescribing practice* or short-term or abbreviat*).tw,kf.<br>27 prescribing practice/<br>28 23 or 24 or 25 or 26 or 27<br>29 22 and 28
**Ovid MEDLINE**	1 exp Percutaneous Coronary Intervention/<br>2 exp drug eluting stent/<br>3 (percutaneous coronary adj3 (interven* or revascular*)).tw,kf.

© 2024 Park DY et al. *JAMA Network Open.*

	4 PCI.ti,ab.<br>5 ((balloon or stent*) adj3 angioplast*).tw,kf.<br>6 ((drug coated or drug eluting or drug releasing) adj3 stent*).tw,kf.<br>7 1 or 2 or 3 or 4 or 5 or 6<br>8 ((antiplatelet or anti-platelet) adj3 (dual or combination) adj3 (intervention* or therap*)).tw,kf.<br>9 DAPT.ti,ab.<br>10 ((Aspirin or Acetylsalicylic acid) and (clopidogrel or plavix or prasugrel or effient or ticagrelor or brilinta or P2Y12 inhibitor*)).tw,kf.<br>11 8 or 9 or 10<br>12 7 and 11 5188<br>13 "duration of therapy"/<br>14 ((duration or length or timing or time or months or reduce* or alterat*) adj3 (dose* or therap* or treatment* or medication* or DAPT or dual anti-platelet)).tw,kf.<br>15 (duration or length or timing or time or month*).ti.<br>16 (de-escalat* or prescribing practice* or short-term or abbreviat*).tw,kf.<br>17 13 or 14 or 15 or 16<br>18 12 and 17<br>19 exp randomized controlled trial/<br>20 single-blind method/<br>21 Double-Blind Method/<br>22 cross-over studies/<br>23 (random* or factorial* or crossover* or placebo* or assign* or allocat* or volunteer*).tw,kf.<br>24 (cross adj1 over).tw,kf.<br>25 ((double or triple or single) adj1 (mask* or blind*)).tw,kf.<br>26 19 or 20 or 21 or 22 or 23 or 24 or 25<br>27 18 and 26
**PubMed**	((((random*[Title/Abstract] OR factorial*[Title/Abstract] OR crossover*[Title/Abstract] OR placebo*[Title/Abstract] OR assign*[Title/Abstract] OR allocat*[Title/Abstract] OR volunteer*[Title/Abstract])) OR (double mask*[Title/Abstract] OR double blind*[Title/Abstract] OR single mask*[Title/Abstract] OR single blind*[Title/Abstract] OR triple blind*[Title/Abstract] OR triple mask*[Title/Abstract] OR cross over[Title/Abstract])) OR (randomized controlled trial[MeSH Terms])) AND (((de-escalat*[Title/Abstract] OR prescribing practice*[Title/Abstract] OR short-term[Title/Abstract] OR abbreviat*[Title/Abstract] OR duration[Title/Abstract] OR length[Title/Abstract] OR timing[Title/Abstract] OR time[Title/Abstract] OR month*[Title/Abstract]) AND ((((Aspirin[Title/Abstract] OR Acetylsalicylic acid[Title/Abstract]) AND (clopidogrel[Title/Abstract] OR plavix[Title/Abstract] OR prasugrel[Title/Abstract] OR effient[Title/Abstract] OR ticagrelor[Title/Abstract] OR brilinta[Title/Abstract] OR P2Y12 inhibitor*[Title/Abstract]))) OR (DAPT[Title/Abstract] OR dual antiplatelet[Title/Abstract] OR dual anti-platelet[Title/Abstract]))) AND (percutaneous coronary interven*[Title/Abstract] OR percutaneous coronary revascular*[Title/Abstract] OR PCI[Title/Abstract] OR balloon angioplast*[Title/Abstract] OR stent* angioplast*[Title/Abstract] OR drug coated stent*[Title/Abstract] OR drug eluting stent*[Title/Abstract] OR drug releasing stent*[Title/Abstract]))
**Scopus**	(TITLE-ABS-KEY ((antiplatelet OR anti-platelet) W/3 (dual OR combination) W/3 (intervention* OR therap*)) OR TITLE-ABS-KEY ((aspirin OR "Acetylsalicylic acid") AND (clopidogrel OR plavix OR prasugrel OR effient OR ticagrelor OR brilinta OR "P2Y12 inhibitor*")) OR TITLE-ABS-KEY (dapt)) AND (TITLE-ABS-KEY ("percutaneous coronary" W/3 (interven* OR revascular*)) OR TITLE-ABS-KEY (pci) OR TITLE-ABS-KEY ((balloon OR stent*) W/3 angioplast*) OR TITLE-ABS-KEY (("drug coated" OR "drug eluting" OR "drug releasing") W/3 stent*)) AND (TITLE-ABS-KEY ((duration OR length OR timing OR time OR months OR reduce* OR alterat*) W/3 (dose* OR therap* OR treatment* OR medication* OR dapt OR "dual anti-platelet")) OR TITLE-ABS-KEY (de-escalat* OR "prescribing practice*" OR short-term OR abbreviat*) OR TITLE (duration OR length OR timing OR time OR month*)) AND (TITLE-ABS-KEY

© 2024 Park DY et al. *JAMA Network Open.*

	(random* OR factorial* OR crossover* OR placebo* OR assign* OR allocat* OR volunteer*) OR TITLE-ABS-KEY (cross W/1 over) OR TITLE-ABS-KEY ((double OR triple OR single) W/1 (mask* OR blind*)))
**Web of Science Core Collection**	#1 TS=("percutaneous coronary" near/3 (interven* or revascular*)) or TS=(PCI) or TS=((balloon or stent*) near/3 angioplast*) or TS=(("drug coated" or "drug eluting" or "drug releasing") near/3 stent*)<br>#2 TS=((antiplatelet or anti-platelet) near/3 (dual or combination) near/3 (intervention* or therap*)) or TS=((Aspirin or "Acetylsalicylic acid") and (clopidogrel or plavix or prasugrel or effient or ticagrelor or brilinta or "P2Y12 inhibitor*")) or TS=(DAPT)<br>#3 TS=((duration or length or timing or time or months or reduce* or alterat*) near/3 (dose* or therap* or treatment* or medication* or DAPT or "dual anti-platelet")) OR TS=(de-escalat* or "prescribing practice*" or short-term or abbreviat*) OR TI=(duration or length or timing or time or month*)<br>#4 TS=(random* or factorial* or crossover* or placebo* or assign* or allocat* or volunteer*) OR TS=(cross near/1 over) OR TS=((double or triple or single) near/1 (mask* or blind*))<br>#5 #1 and #2 and #3 and #4

© 2024 Park DY et al. *JAMA Network Open.*

eTable 2. Baseline Demographics of the Selected Trials

<table>
<tr><th>Abbreviated/Standard[a], %</th><th>HOST-IDEA</th><th>MASTER DAPT</th><th>TICO</th><th>SMART-CHOICE</th><th>TWILIGHT</th><th>STOPDAPT-2</th><th>REDUCE</th></tr>
<tr><td>Age, year, mean</td><td>65.6/65.9</td><td>76.1/76.0</td><td>61.0/61.0</td><td>64.6/64.4</td><td>65.2/65.1</td><td>68.1/69.1</td><td>61.0/60.0</td></tr>
<tr><td>Female</td><td>27.0/25.2</td><td>30.7/30.8</td><td>21.0/20.0</td><td>27.3/25.8</td><td>23.8/23.9</td><td>21.1/23.5</td><td>17.4/22.7</td></tr>
<tr><td>BMI, kg/m^2, mean</td><td>NA</td><td>27.3/27.4</td><td>24.9/24.9</td><td>24..5/24.7</td><td>28.6/28.5</td><td>24.4/24.2</td><td>NA</td></tr>
<tr><td>Diabetes mellitus</td><td>40.5/37.4</td><td>32.9/34.3</td><td>27.0/27.0</td><td>38.2/36.8</td><td>9.4/10.5</td><td>39.0/38.0</td><td>21.6/19.5</td></tr>
<tr><td>Hypertension</td><td>73.3/73.5</td><td>76.9/78.2</td><td>50.0/51.0</td><td>61.6/61.3</td><td>72.6/72.2</td><td>73.7/74.0</td><td>50.7/50.7</td></tr>
<tr><td>Dyslipidemia</td><td>81.2/80.1</td><td>67.2/68.1</td><td>61.0/60.0</td><td>45.1/45.5</td><td>60.7/60.2</td><td>74.4/74.8</td><td>46.3/44.9</td></tr>
<tr><td>Current smoking</td><td>NA</td><td>10.0/8.1</td><td>NA</td><td>28.4/24.5</td><td>20.4/23.1</td><td>26.6/20.6</td><td>42.1/42.7</td></tr>
<tr><td>Family history</td><td>NA</td><td>NA</td><td>NA</td><td>NA</td><td>NA</td><td>NA</td><td>35.0/36.0</td></tr>
<tr><td>Chronic kidney disease</td><td>11.0/10.5</td><td>18.2/20.1</td><td>19.0/22.0</td><td>2.9/3.5</td><td>16.8/16.7</td><td>5.5/5.6</td><td>NA</td></tr>
<tr><td>Peripheral vascular disease</td><td>1.9/1.9</td><td>NA</td><td>NA</td><td>NA</td><td>6.9/6.8</td><td>6.4/6.6</td><td>NA</td></tr>
<tr><td>Heart failure</td><td>NA</td><td>NA</td><td>NA</td><td>NA</td><td>NA</td><td>7.7/7.1</td><td>NA</td></tr>
<tr><td>Prior myocardial infarction</td><td>4.5/4.2</td><td>18.9/18.8</td><td>4.0/3.0</td><td>4.1/4.3</td><td>28.7/28.6</td><td>13.8/13.2</td><td>NA</td></tr>
<tr><td>Prior PCI</td><td rowspan="2">13.2/14.1</td><td>25.9/26.0</td><td>NA</td><td>11.5/11.8</td><td>42.3/42.0</td><td>33.5/35.1</td><td>11.7/9.8</td></tr>
<tr><td>Prior CABG</td><td>7.4/7.5</td><td>1.0/1.0</td><td>NA</td><td>10.2/9.8</td><td>1.1/2.8</td><td>2.8/2.8</td></tr>
<tr><td>Prior stroke</td><td>6.9/6.3</td><td>NA</td><td>4.0/4.0</td><td>6.6/6.8</td><td>NA</td><td>NA</td><td>1.5/2.0</td></tr>
<tr><td>Prior bleeding</td><td>NA</td><td>7.2/6.8</td><td>NA</td><td>NA</td><td>0.9/0.9</td><td>1.3/1.9</td><td>NA</td></tr>
<tr><td>LVEF, %, mean</td><td>58.2/58.6</td><td>53.5/53.0</td><td>NA</td><td>60.0/59.9</td><td>NA</td><td>59.8/59.7</td><td>NA</td></tr>
<tr><td>Multivessel disease</td><td>51.8/51.7</td><td>NA</td><td>55.0/56.0</td><td>50.1/49.0</td><td>63.9/61.6</td><td>NA</td><td>NA</td></tr>
<tr><td colspan="8">Clinical presentation</td></tr>
<tr><td>Silent ischemia</td><td rowspan="2">43.4/46.3</td><td>10.7/12.0</td><td>NA</td><td>NA</td><td>6.6/6.3</td><td>NA</td><td>NA</td></tr>
<tr><td>Stable angina</td><td>40.2/40.6</td><td>NA</td><td>41.8/41.8</td><td>29.5/28.0</td><td>62.3/61.4</td><td>NA</td></tr>
<tr><td>Unstable angina</td><td>35.7/34.7</td><td>11.3/11.4</td><td>29.0/32.0</td><td>31.2/32.8</td><td>35.1/34.9</td><td>12.9/14.2</td><td>15.2/13.8</td></tr>
<tr><td>NSTEMI</td><td>20.9/19.0</td><td>25.9/24.4</td><td>35.0/32.0</td><td>16.0/15.4</td><td>28.8/30.8</td><td>5.4/6.6</td><td>35.6/41.0</td></tr>
<tr><td>STEMI</td><td>NA</td><td>11.9/11.6</td><td>36.0/36.0</td><td>11.0/10.0</td><td>NA</td><td>19.4/17.9</td><td>49.3/45.2</td></tr>
</table>

© 2024 Park DY et al. *JAMA Network Open.*

eTable 2. Baseline Demographics of the Selected Trials (continued)

**Abbreviated/Standard[a], %**	**GLOBAL LEADERS**	**SMART-DATE**	**IVUS-XPL**	**ISAR-SAFE**	**I-LOVE-IT 2**	**RESET**	**EXCELLENT**
Age, year, mean	64.9/64.8	62.0/62.2	63.0/64.0	67.2/67.2	60.4/60.0	62.4/62.4	63.0/62.4
Female	24.0/23.5	25.1/24.1	33.0/30.0	19.3/19.5	32.8/31.3	35.6/37.1	34.9/36.1
BMI, kg/m^2, mean	NA	24.3/24.5	24.8/24.6	27.2/27.5	25.1/25.3	25.0/24.9	24.9/25.1
Diabetes mellitus	24.3/23.7	26.9/28.1	36.0/37.0	24.8/24.2	23.2/22.1	29.8/28.8	37.7/38.6
Hypertension	72.5/72.3	49.9/48.7	NA	90.1/91.5	61.0/64.8	62.3/61.4	72.7/73.8
Dyslipidemia	63.3/65.3	24.2/25.2	68.0/65.0	87.5/87.4	25.3/23.4	57.7/59.9	75.2/76.3
Current smoking	2.6/2.6	38.0/40.1	25.0/24.0	14.6/15.3	36.6/38.3	25.2/22.8	27.4/25.8
Family history	NA	NA	NA	NA	6.3/5.1	NA	NA
Chronic kidney disease	13.4/13.1	1.0/0.5	NA	NA	NA	NA	0.8/1.2
Peripheral vascular disease	6.7/7.9	NA	NA	NA	1.4/1.1	NA	NA
Heart failure	NA	NA	NA	NA	NA	11.3/11.8	0.6/0.7
Prior myocardial infarction	22.9/23.6	2.3/1.7	5.0/4.0	25.9/24.5	17.2/15.8	1.8/1.6	6.5/3.7
Prior PCI	32.6/33.9	4.9/3.9	10.0/10.0	NA	8.5/6.5	3.5/3.0	9.3/8.6
Prior CABG	NA	NA	3.0/2.0	7.7/7.5	0.4/0.4	0.2/0.6	NA
Prior stroke	NA	3.9/4.4	NA	NA	9.2/9.5	NA	6.5/6.7
Prior bleeding	0.7/0.6	NA	NA	NA	NA	NA	NA
LVEF, %, mean	55.1/55.3	55.5/55.4	62.3/63.1	NA	60.8/60.3	64.2/63.9	61.0/61.6
Multivessel disease	NA	43.6/46.6	NA	NA	NA	43.1/42.9	NA
**Clinical presentation**							
Silent ischemia	NA	NA	NA	10.9/11.3	3.0/4.0	NA	48.9/48.0
Stable angina	48.9/49.9	NA	51.0/51.0	48.6/47.8	14.3/15.1	44.5/46.3	
Unstable angina	12.9/13.2	31.0/3.7	34.0/33.0	21.5/21.9	58.0/56.5	40.8/39.9	48.5/48.4
NSTEMI	20.0/19.4	31.5/31.4	15/16	10.4/10.1	13.4/13.7	14.7/13.8	
STEMI	18.2/17.5	37.5/37.9		7.9/8.3	11.3/10.7		2.6/3.6

[a]The data entry on the left ("abbreviated") refers to the experimental group in which the duration of dual antiplatelet therapy was shortened. The data entry on the right ("standard") refers to the control group in which the duration of dual antiplatelet therapy was continued for standard duration according to current guidelines.

Abbreviations: BMI, body mass index; CABG, coronary artery bypass graft; LVEF, left ventricular ejection fraction; NSTEMI, non-ST-elevation myocardial infarction; PCI, percutaneous coronary intervention; STEMI, ST-elevation myocardial infarction

© 2024 Park DY et al. *JAMA Network Open.*

eTable 3. Definition of Outcomes Used in the Trials

Trial	Net Adverse Clinical Events	Major Adverse Cardiovascular Events	Bleeding
**HOST-IDEA**	Cardiac death, target vessel MI, clinically driven TLR, stent thrombosis, BARC type 3 or 5 bleeding	NA[a]	NA[a]
**MASTER DAPT**	MACE, BARC type 3 or 5 bleeding	All-cause mortality, MI, stroke	Major or nonmajor clinically relevant bleeding
**TICO**	MACE, TIMI major bleeding	All-cause mortality, MI, stent thrombosis, stroke, TVR	TIMI major bleeding
**SMART-CHOICE**	NA[a]	All-cause mortality, MI, stroke	BARC type 2-5 bleeding
**TWILIGHT**	NA[a]	All-cause mortality, MI, stroke	BARC type 2, 3, or 5 bleeding
**STOPDAPT-2**	Cardiovascular death, MI, stent thrombosis, stroke, any bleeding	NA[a]	NA[a]
**REDUCE**	All-cause mortality, MI, stent thrombosis, stroke, TVR, BARC type 2, 3, or 5 bleeding	NA[a]	NA[a]
**GLOBAL LEADERS**	NA[a]	All-cause mortality, MI, stroke, urgent TVR	BARC type 3 or 5 bleeding
**SMART-DATE**	NA[a]	All-cause mortality, MI, stroke	NA[a]
**IVUS-XPL**	Cardiac death, MI, stroke, TIMI major bleeding	Cardiac death, MI, revascularization	TIMI major bleeding
**ISAR-SAFE**	All-cause mortality, MI, stent thrombosis, stroke, TIMI major bleeding	NA[a]	NA[a]
**I-LOVE-IT 2**	All-cause mortality, MI, stroke, BARC type 3-5 bleeding	Cardiac death, target vessel MI, clinically indicated TLR	NA[a]
**RESET**	Cardiac death, MI, stent thrombosis, ischemia-driven TVR, TIMI major bleeding	NA[a]	NA[a]
**EXCELLENT**	NA[a]	All-cause mortality, MI, stroke, revascularization	NA[a]

[a]Not applicable because this outcome was not reported in the older adult population

Abbreviations: BARC, Bleeding Academic Research Consortium; MI, myocardial infarction; TIMI, Thrombolysis In Myocardial Infarction; TLR, target lesion revascularization; TVR, target vessel revascularization

© 2024 Park DY et al. *JAMA Network Open.*

eTable 4. Risk of Bias in the Selected Studies Using the Cochrane Risk Assessment Tool

Trial	Random Sequence Generation (Selection Bias)	Allocation Concealment (Selection Bias)	Blinding of Participants and Personnel (Performance Bias)	Blinding of Outcome Assessment (Detection Bias)	Incomplete Outcome Data (Attrition Bias)	Selective Reporting (Reporting Bias)	Other Bias
**HOST-IDEA**	Low	Low	High	Low	Low	Low	Unclear
**MASTER DAPT**	Low	Low	Low	Low	Low	Low	Unclear
**TICO**	Low	Low	High	Low	Low	Low	Unclear
**SMART CHOICE**	Low	Low	High	Low	Low	Low	Unclear
**TWILIGHT**	Low	Low	Low	Low	Low	Low	Unclear
**STOPDAPT-2**	Low	Unclear	High	Low	Low	Low	Unclear
**REDUCE**	Low	Low	High	Low	Low	Low	High
**GLOBAL LEADERS**	Low	Low	Low	Low	Low	Low	Unclear
**SMART-DATE**	Low	Low	High	Low	Low	Low	Unclear
**IVUS-XPL**	Low	Low	High	Low	Low	Low	High
**ISAR-SAFE**	Low	Low	Low	Low	Low	Low	Unclear
**I-LOVE-IT 2**	Low	Unclear	Unclear	Unclear	Low	Low	High
**RESET**	Low	Low	High	Unclear	Low	Low	Unclear
**EXCELLENT**	Low	Low	High	Low	Low	Low	High
**Overall Bias**	Low	Low	High	Low	Low	Low	Unclear

© 2024 Park DY et al. *JAMA Network Open.*

eTable 5. Grading of Recommendations, Assessment, Development and Evaluations (GRADE) Criteria for Each Outcome

Outcome	Trials	Risk of Bias	Imprecision	Inconsistency	Indirectness	Publication Bias	Certainty
Net adverse clinical events	9	Low	High	Low	Low	Low	⊕⊕⊕◯
Major adverse cardiovascular events	9	Low	Moderate	Low	Low	Low	⊕⊕⊕⊕
Bleeding	6	Low	Low	Moderate	Low	Low	⊕⊕⊕⊕

© 2024 Park DY et al. *JAMA Network Open*.

eTable 6. Heterogeneity Assessment in Network Meta-Analysis

Outcome	τ^2	I^2
**Net adverse clinical events**	0.0782	46.7%
**Major adverse cardiovascular events**	0.0015	2.6%
**Bleeding**	0	0%

© 2024 Park DY et al. *JAMA Network Open.*

eTable 7. Node-Splitting Analysis of Inconsistency for Each Outcome

Outcome	Comparison	*K*[a]	Prop[b]	*NMA*[c]	Direct[d]	Indirect[e]	*Z*-value[f]	*P*-value[g]
**Net adverse clinical events**	12 months versus 1 month	1	0.42	0.5164	0.8085	0.3077	0.83	0.4084
	3 months versus 1 month	0	0	0.2561	NA	0.2561	NA	NA
	6 months versus 1 month	1	0.75	0.2359	0.1092	0.6099	-0.83	0.4084
	12 months versus 3 months	4	1.00	0.2603	0.2603	NA	NA	NA
	12 months versus 6 months	3	0.84	0.2805	0.1985	0.6993	-0.83	0.4084
	3 months versus 6 months	0	0	0.0202	NA	0.0202	NA	NA
**Major adverse cardiovascular events**	12 months versus 1 month	1	0.63	0.2408	0.2739	0.1844	0.34	0.7322
	3 months versus 1 month	0	0	0.1756	NA	0.1756	NA	NA
	6 months versus 1 month	1	0.70	0.1236	0.0969	0.1863	-0.34	0.7322
	12 months versus 3 months	3	1.00	0.0652	0.0652	NA	NA	NA
	12 months versus 6 months	4	0.67	0.1172	0.0875	0.1770	-0.34	0.7322
	3 months versus 6 months	0	0	0.0520	NA	0.0520	NA	NA
**Bleeding**	12 months versus 1 month	1	0.86	0.2462	0.1861	0.6217	-0.68	0.4949
	3 months versus 1 month	0	0	-0.3207	NA	-0.3207	NA	NA
	6 months versus 1 month	1	0.96	0.3807	0.3961	-0.0395	0.68	0.4949
	12 months versus 3 months	3	1.00	0.5669	0.5669	NA	NA	NA
	12 months versus 6 months	1	0.17	-0.1345	0.2256	-0.2100	0.68	0.4949
	3 months versus 6 months	0	0	-0.7014	NA	-0.7014	NA	NA

[a]Number of studies providing direct evidence
[b]Proportion of direct evidence
[c]Estimated treatment effect (risk ratio) in network meta-analysis
[d]Estimated treatment effect (risk ratio) derived from direct evidence
[e]Estimated treatment effect (risk ratio) derived from indirect evidence
[f]Z-value of test for disagreement between direct and indirect evidence
[g]P-value of test for disagreement between direct and indirect evidence
Abbreviations: NMA, network meta-analysis treatment effect; prop, proportion

© 2024 Park DY et al. *JAMA Network Open.*

eTable 8. Test of Inconsistency Between Designs

Outcome	*P*-value		
	**Within Designs**	**Between Designs**	**Total**
**Net adverse clinical events**	0.0891	0.1904	0.0806
**Major adverse cardiovascular events**	0.3032	0.7223	0.4058
**Bleeding**	0.4737	0.4949	0.5807

© 2024 Park DY et al. *JAMA Network Open.*

eTable 9. P-Scores of Each Duration of Dual Antiplatelet Therapy

Outcome	1 month	3 months	6 months	12 months
**Net adverse clinical events**	0.8445	0.5373	0.5355	0.0827
**Major adverse cardiovascular events**	0.8866	0.5323	0.4147	0.1664
**Bleeding**	0.6553	0.9658	0.2809	0.098

© 2024 Park DY et al. *JAMA Network Open.*

eTable 10. Cumulative Event Rates at Different Durations of Dual Antiplatelet Therapy

Outcome	12 months	6 months	3 months	1 month
**Net adverse clinical events**	5.40%	6.03%	4.82%	6.40%
**Major adverse cardiovascular events**	5.76%	5.96%	3.98%	7.19%
**Bleeding**	5.84%	8.67%	3.55%	6.16%

© 2024 Park DY et al. *JAMA Network Open.*

eTable 11. Absolute Risk Differences at Shorter Durations of Dual Antiplatelet Therapy

	**6 months**	**3 months**	**1 month**
**Net adverse clinical events**	+0.63%	-0.58%	+1.00%
**Major adverse cardiovascular events**	+0.20%	-1.78%	+1.43%
**Bleeding**	+2.83%	-2.29%	+0.32%

© 2024 Park DY et al. *JAMA Network Open.*

eTable 12. Sensitivity Analysis Only Including Trials That Defined Older Adults as Age ≥65 Years

**Net adverse clinical events**[a] (6 trials included)	**1 month**			
	NA	**3 months**		
	NA	0.76 (0.46-1.26)	**6 months**	
	NA	0.64 (0.46-0.91)	0.85 (0.58-1.24)	**12 months**
**Major adverse cardiovascular events**[a] (7 trials included)	**1 month**			
	NA	**3 months**		
	NA	1.01 (0.66-1.56)	**6 months**	
	NA	0.92 (0.69-1.22)	0.91 (0.66-1.26)	**12 months**
**Bleeding**[a] (4 trials included)	**1 month**			
	NA	**3 months**		
	NA	0.71 (0.22-2.27)	**6 months**	
	NA	0.57 (0.45-0.71)	0.80 (0.26-2.49)	**12 months**

[a]This sensitivity analysis included trials that provided results in older adults ≥65 years-old, as well as one trial using a cutoff of >67.2 years (ISAR-SAFE[1]) and one trial using a cutoff of >65 years (HOST-IDEA[2]). The duration of dual antiplatelet therapy at the rightmost column serves as the reference group for the respective column. Values are not available for 1 month of dual antiplatelet therapy because of the absence of trial that set its experimental group as 1 month of dual antiplatelet therapy and defined older adults as age ≥65 years.

© 2024 Park DY et al. *JAMA Network Open.*

eTable 13. Sensitivity Analysis Only Including Trials That Reported Major Bleeding in Older Adults

**Major bleeding**[a] (3 trials included)	**1 month**			
	1.74 (0.77-3.94)	**3 months**		
	1.04 (0.30-3.55)	0.60 (0.16-2.25)	**6 months**	
	0.83 (0.52-1.32)	0.48 (0.24-0.94)	0.80 (0.26-2.49)	**12 months**

[a]The duration of dual antiplatelet therapy at the rightmost column serves as the reference group for the respective column.

© 2024 Park DY et al. *JAMA Network Open.*

eFigure. Node-Splitting Analysis of Inconsistency for Each Outcome

Net adverse clinical events

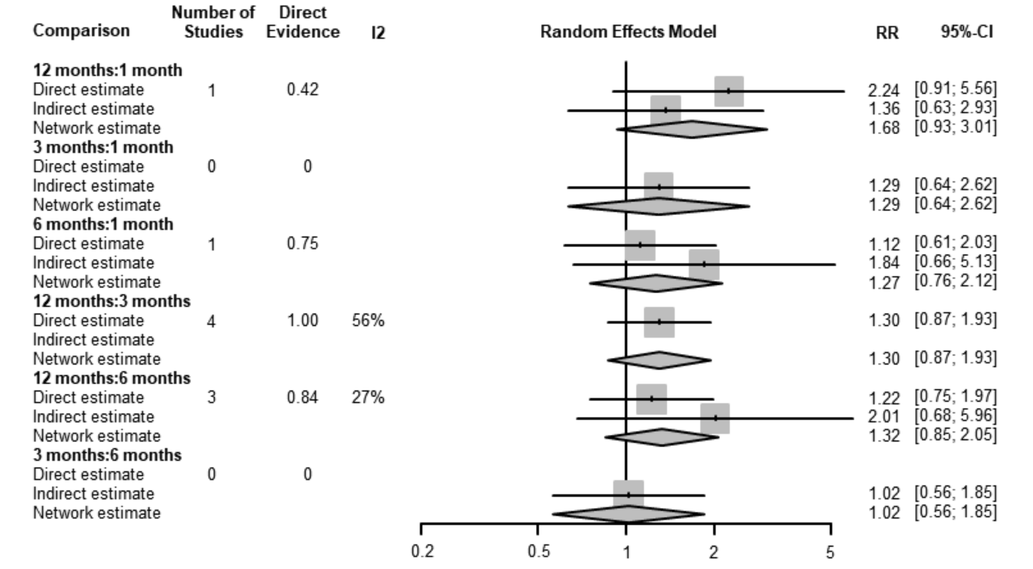

Major adverse cardiovascular events

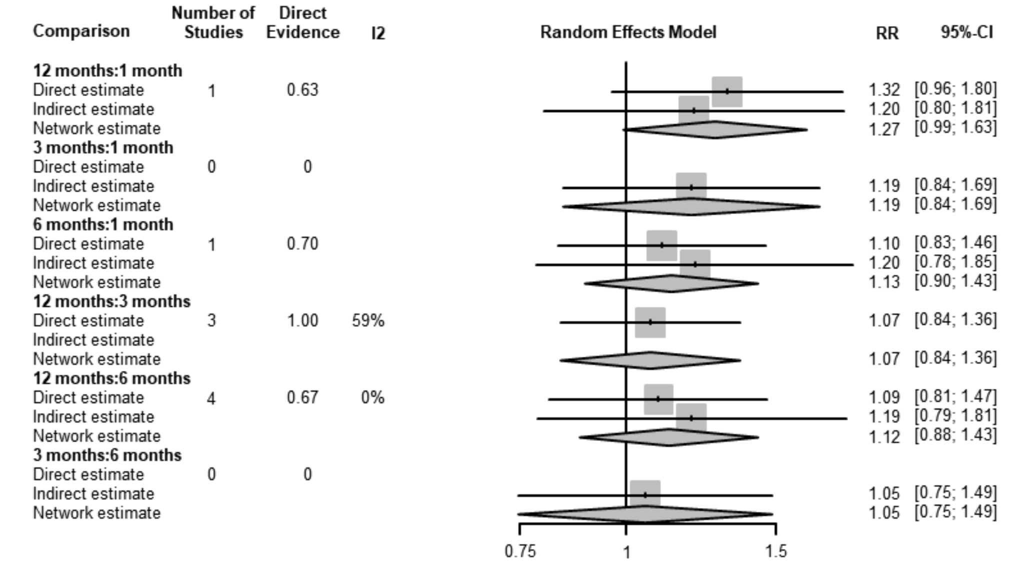

© 2024 Park DY et al. *JAMA Network Open.*

**Bleeding**	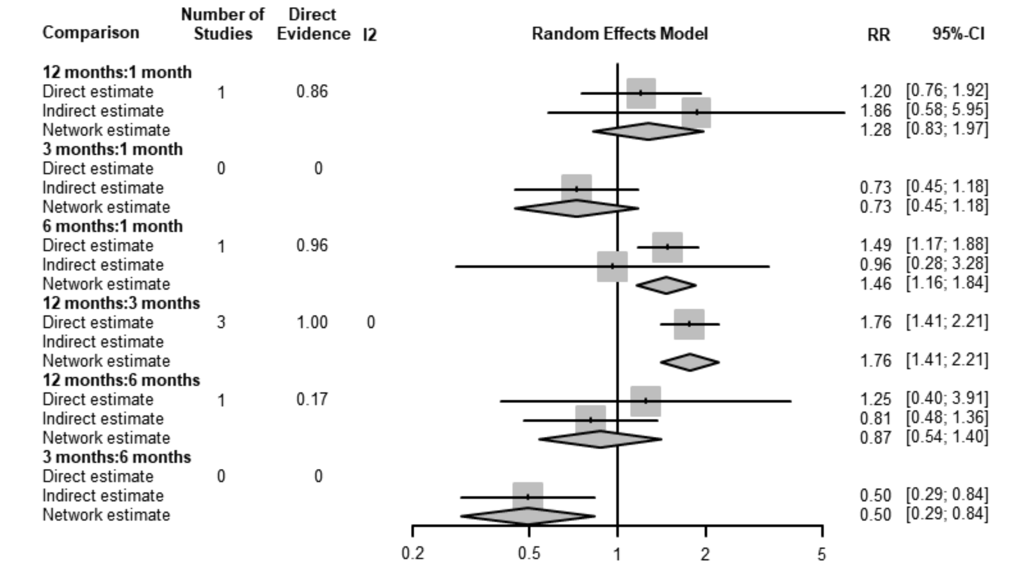

Abbreviation: CI, confidence interval; RR, risk ratio

© 2024 Park DY et al. *JAMA Network Open*.

eReferences.

1. Schulz-Schupke S, Byrne RA, Ten Berg JM, et al. ISAR-SAFE: a randomized, double-blind, placebo-controlled trial of 6 vs. 12 months of clopidogrel therapy after drug-eluting stenting. *Eur Heart J*. May 21 2015;36(20):1252-63. doi:10.1093/eurheartj/ehu523

2. Han JK, Hwang D, Yang S, et al. Comparison of 3- to 6-Month Versus 12-Month Dual Antiplatelet Therapy After Coronary Intervention Using the Contemporary Drug-Eluting Stents With Ultrathin Struts: The HOST-IDEA Randomized Clinical Trial. *Circulation*. May 2 2023;147(18):1358-1368. doi:10.1161/CIRCULATIONAHA.123.064264

© 2024 Park DY et al. *JAMA Network Open.*
