# Supplementary material for: Shorter Dual Antiplatelet Therapy for Older Adults After Percutaneous Coronary Intervention: A Systematic Review and Network Meta-Analysis
Source: JAMA Netw Open. 2024 Mar 28;7(3):e244000. doi: 10.1001/jamanetworkopen.2024.4000 (PMC10979312; doi:10.1001/jamanetworkopen.2024.4000)
Supplement: Supplement 2. — Data Sharing Statement [file jamanetwopen-e244000-s002.pdf]

Data Sharing Statement

Park. Shorter Dual Antiplatelet Therapy for Older Adults After Percutaneous Coronary Intervention. *JAMA Netw Open*. Published March 28, 2024. doi:10.1001/jamanetworkopen.2024.4000

Data
Data available: No
